# Understanding the epidemiological characteristics of the primary healthcare corporation-based COVID-19 swabbed persons in Qatar, 2020

**DOI:** 10.5339/qmj.2022.23

**Published:** 2022-07-11

**Authors:** Mariam Ali Abdulmalik, Mohamed Ghaith Al-Kuwari, Ahmad Haj Bakri, Samya Ahmad Al Abdulla, Mujeeb Chettiyam Kandy, Maha Yousef Abdulla, John Michael Gibb

**Affiliations:** Primary Health Care Corporation, Doha, Qatar E-mail: abakri@phcc.gov.qa

**Keywords:** Primary Health Care, COVID-19, Epidemiology, Qatar

## Abstract

Background: In March 2020, Qatar started reporting increased numbers of severe acute respiratory syndrome coronavirus 2 (SARS-CoV-2), causing coronavirus disease 2019 (COVID-19). National preventive measures were implemented, and a testing plan was developed to respond to the pandemic with the Primary Health Care Corporation (PHCC) as the central element. PHCC is the main public primary healthcare provider in Qatar and it operates in 27 health centers with around 1.4 million registered individuals as of January 1, 2020. The latter population was distributed across four main nationality groups; Middle Eastern and North African (51.5%), Asian (41.2%), African (2.4%), and others (5.1%). At the primary healthcare level in Qatar, this study describes the epidemiological characteristics of individuals registered at PHCC who had contracted COVID-19 in 2020 during the first wave before the vaccination phase and examines the factors associated with the positivity rate.

Methods: Retrospective data analysis was conducted for persons screened for SARS-CoV-2 in primary healthcare health centers in Qatar between March 11 and December 31, 2020. The study analyzed the demographic characteristics of the tested persons and noncommunicable disease burden, positivity rate by month, nationality, and age-group, and the factors associated with the positivity rate.

Results: Between March 11 and December 31, 2020, PHCC tested 379,247 persons for SARS-CoV-2, with a median age (IQR) of 32 (21–42) years. Of these, 57.0% were from the Middle East and North Africa, and 32.5% were originally from Asia. Overall, 10.9% had diabetes mellitus and 11.3% had hypertension. The epidemiological curve showed a steep increase in the positivity rate from March till May 2020, at the highest rate of 37.5% in May 2020. The highest positivity rate was observed among Asian males at 15.7%. The positivity rate was the lowest among the age-group aged 60 years and above. It was almost the same among the tested persons for SARS-CoV-2 in the three main age groups (0–18, 19–39, 40–59) at 10.1%, 12.3%, and 12.2%, respectively. In a multi regression model, being a male was associated with a higher risk (OR 1.15; 95% CI 1.13–1.17). Asians were at higher risk than those originally from the Middle East and North Africa (OR 1.29; 95% CI 1.27–1.32). COVID-19 infection was higher among those presenting clinical symptoms than asymptomatic individuals (OR. 4.52; 95% CI 4.42–4.64).

Conclusion: The epidemic among the PHCC-registered population predominantly affected younger ages and males, namely, coming from Asia. At the primary healthcare level, the COVID-19 infection rate was higher among those who presented with clinical symptoms. The lowest positivity rate among individuals >60 years may reflect the effectiveness of public health measures related to the high-risk group. Scaled-up testing at the primary healthcare level helped to detect more cases during the peak of the first wave and was reflected in a steady increase in the positivity rate flattened later due to the established public health measures.

## Introduction

In December 2019, a cluster of patients with unknown causes of pneumonia was reported in Wuhan, China.^1,2^ The samples’ viral genetic sequencing indicated a novel coronavirus.^1^ This novel coronavirus, severe acute respiratory syndrome coronavirus 2 (SARS-CoV-2), was isolated as the causative organism, and the subsequent disease was called COVID-19.^1,3,4^ It was presumed initially to be transmitted from animals to humans; however, the virus has since spread rapidly globally through human to human transmission.^5–7^ As of December 29, 2020, approximately 79 million cases and over 1.7 million deaths have been reported globally.^8^

The published epidemiological studies across several populations demonstrate substantial differences in the infection severity and rates and case-fatality rates.^9^ Hence, analyzing the transmission patterns among a population with unique demographics can improve the knowledge of disease dynamics.

Qatar is located in the Middle East peninsula and has borders with Saudi Arabia. Qatar's estimated population in July 2020 was almost 2.4 million. It has a unique population demographic profile in which the expatriate workforce constitutes almost 88% of the population.^10^ The expatriate workforce nature in Qatar influences the male-to-female ratio with 75% males to 25% females. Also, the population pyramid is condensed into the age groups of 25–40 years, namely among males.^11^ Evidence from other countries demonstrated that COVID-19 disproportionally affects males with poorer outcomes among the older age groups.^12,13^

Increased population movement and mobility made it possible for COVID-19 to spread more easily and faster.^14^ In Qatar, a general restriction on all incoming international flights was implemented on March 31, 2020. The latter helped halt almost all visitors or residents from entering the country.

In March 2020, Qatar started reporting increased numbers of COVID-19 positive cases. At that stage, national restrictions were implemented. The Ministry of Public Health in Qatar developed an emergency action plan to respond to the COVID-19 outbreak, with the Primary Health Care Corporation (PHCC) as the main player in the pandemic's response.

**Figure 1.**
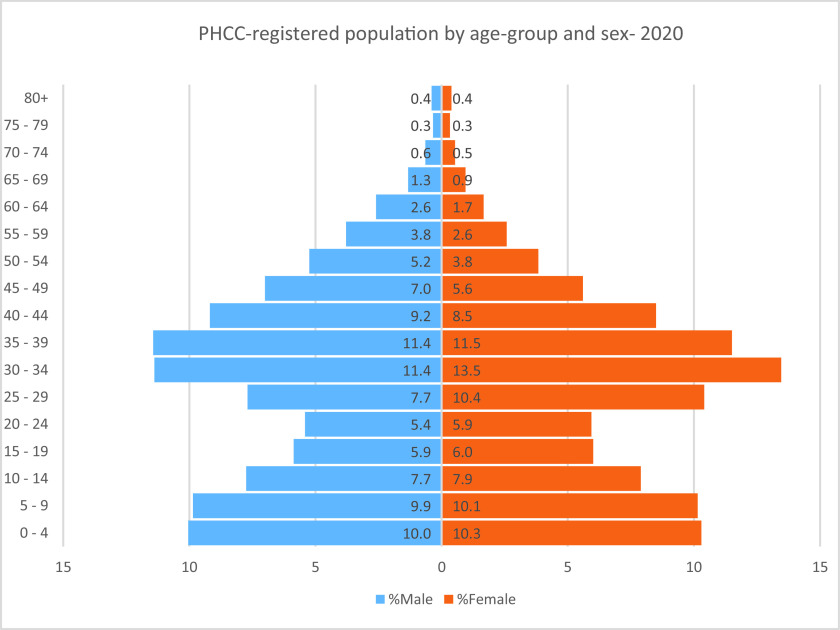
Primary Health Care Corporation (PHCC)-registered population distributed by five-year age-group and sex as of December 31, 2020.Source: Strategy Planning and Health Intelligence. Primary Health Care Corporation. Doha. 2021

PHCC, the main public primary care provider for families in Qatar, serves 1.4 million people throughout 27 primary healthcare centers covering all three main regions of the country (central, northern, and western).^15^ The latter population was distributed across four main nationality groups; Middle Eastern and North African (51.5%), Asian (41.2%), African (2.4%), and other Europeans, Americans, Australians, and New Zealanders (5.1%) as of January 1, 2020, based on an internal assessment conducted by the Strategy Planning and Health Intelligence Directorate at the PHCC. The population pyramid of the PHCC-registered population in 2020 reflected an equal distribution between males and females across the five-year age groups, as shown in Figure 1 since most manual craft workers are registered with Qatar Red Crescent health centers for their primary healthcare services.^16^

PHCC responded rapidly to the pandemic by opening the first COVID-19 center for testing and holding screening on March 12, 2020, for its registered population. By the end of March 2020, the PHCC scaled-up its testing capacity at the community level for the PHCC-registered population by designating three additional health centers for testing and holding and allocating at least one testing room in the remaining 23 health centers.^17^

This study defines the epidemiological characteristics of COVID-19 among the PHCC-registered population in Qatar in 2020 during the first wave before vaccine administration. It examines the factors associated with the infectious rate. Understanding the epidemiological characteristics associated with COVID-19 infection at the PHCC level in Qatar will be beneficial for understanding the epidemiology in countries with unique demographic characteristics.

## Methods

By March 11, 2020, PHC had four designated health centers for COVID-19 testing and at least one allocated room in each of the remaining 23 health centers for COVID-19 testing. All COVID-19 testing in Qatar was performed by Hamad Medical Corporation central laboratory, the leading public secondary/tertiary care provider in Qatar, and provides more than 85% of the inpatient bed capacity in the country.^18^ The test results are shared with the PHCC on an hourly basis through a joint electronic medical record system between the two entities. The Ministry of Public Health designed and implemented an effective contract tracing system. For each person who tested positive, a thorough contact tracing was conducted by trained staff at the Ministry of Public Health.^18^

Between March 11 and December 31, 2020, persons who were tested at PHCC testing sites were primarily those presenting with influenza-like symptoms suggesting COVID-19 illness and others who had been in contact with COVID-19 patients. Nasopharyngeal and throat swabs were collected from the latter. To detect SARS-CoV-2 infection, real-time ribonucleic acid polymerase chain reaction, RNA-PCR was utilized using TaqPath COVID-19 combo kit (Thermo Fisher Scientific, Waltham, Massachusetts, USA) or Cobas SARS-CoV-2 test (Roche Diagnostics, Rotkreuz, Switzerland). These tests are proven to be highly sensitive and specific.^19,20^

The nationality of each tested person was established based on the official identification state card issued for all residents and nationals in Qatar. Nationalities were grouped into four main groups based on the demographic distribution of ethnicities in Qatar for analysis purposes (Middle Eastern and North African, Asian, African, and others for Europeans, Americans, Australians, and New Zealanders). Demographic characteristics and noncommunicable disease burden among the tested persons were derived from electronic medical records. The age of the tested persons was categorized into four main groups (0–18, 19–39, 40–59, and 60+). The latter age-group categorizations aligned with the National Health Strategy 2018–2022 age-group categories.

Throughout the study period, a timeline of the positivity rate was created to study the epidemic trend at the primary healthcare level. The positivity rate was calculated among the different grouped nationalities, age groups, and persons presenting with symptoms. Multivariable logistic regression was used to determine the effect of age, symptoms presentation, and grouped nationality on capturing COVID-19 infection.

After securing all the necessary approvals from the PHCC scientific committee in response to the national and global public health emergency, this study was conducted. There was no direct patient or public involvement, yet the key elements of the data were shared daily with the public.

## Results

Between March 11 and December 31, 2020, 379,204 persons (182,621 females and 196,583 males), corresponding to 48.2% females and 51.8% males, were tested for SARS-CoV-2 at the PHCC health centers, with a median age (IQR) of 32 (21–42) years. Around 48.2% of those tested persons were in the age-group of 19–39 years, followed by 24.0% and 21.8% of them falling into the age-group of 40–59 years and 0–18 years, respectively, Table 1. Of the tested persons for SARS-CoV-2, 57.0% were originally from the Middle East and North Africa, followed by 32.5% originally from Asia, as indicated in Table 1.

The main reason for swabbing was clinical suspicion, with 27.0% of persons being swabbed due to influenza-like symptoms and severe acute respiratory infection. Random swabbing surveys among the clusters of high-risk populations and contact tracing swabbing for persons identified as in contact with positive COVID-19 cases based on the criteria published by the US Centers for Disease Control and Prevention were among the main reasons for swabbing with 12.4% and 8.7%, respectively, as shown in Table 1.

Among the persons tested for SARS-CoV-2 at the PHCC testing sites, the most common comorbidities were diabetes mellitus (10.9%), hypertension (11.3%), asthma and COPD (9.0%), and cardiovascular diseases (2.3%). Diabetes mellitus was slightly higher among males than females (11.9% vs. 9.8%).

**Figure 2.**
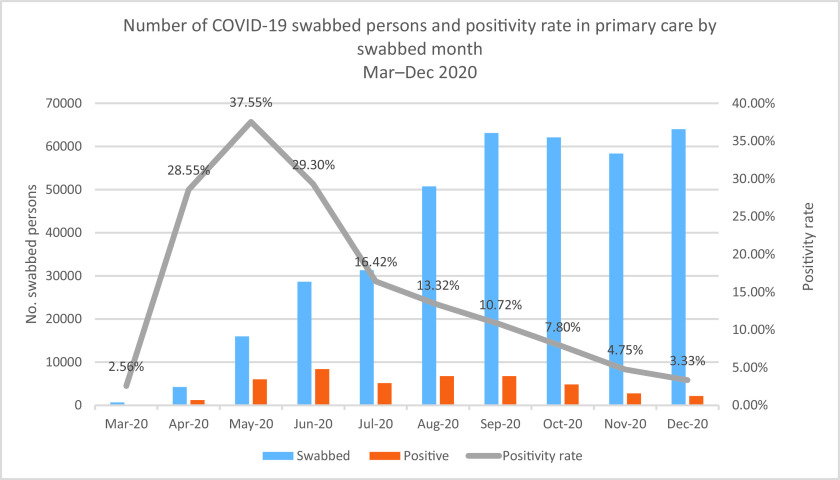
Number of persons swabbed for COVID-19 at the PHCC health centers and positivity rate by month

At the beginning of March 2020, when the COVID-19 cases started rising in Qatar, the PHCC responded to the outbreak by establishing COVID-19 testing sites and holding centers. The positivity rate was calculated per month, as demonstrated in Figure 2. The epidemiological curve showed a steep increase in the positivity rate between March and May 2020. Then there was a decrease in the positivity rate as of June, reaching its lowest rate in December 2020 at 3.33%.

The grouped nationalities with the highest positivity rates were Asian (13.9%), Middle Eastern and North African (11.1%), and African (10.0%), as shown in Table 2. Asian males had the highest positivity rate at 15.7%. The positivity rates were almost the same among the tested persons for SARS-CoV-2 in the main three age groups (0–18, 19–39, 40–59) at 10.1%, 12.3%, and 12.2%, respectively. Health centers located in the western region of Qatar had the highest positivity rate at 12.3% than the remaining health centers located in the central and northern regions at 11.2% and 11.4, respectively. The positivity rate was the highest among persons who presented with the clinical symptoms of influenza-like illness and severe acute respiratory infection at 18.3%, as illustrated in Table 2.

In a multivariable logistic regression model, sex, nationality group, age groups, and symptom presentation were examined as exposure factors to catching COVID-19 infection. Sex was found to affect that being a male was associated with a higher risk (OR 1.15; 95% CI 1.13–1.17). Nationality group affected catching COVID-19 infection, with persons originally from Asia being at higher risk than those originally from the Middle East and North Africa (OR 1.29; 95% CI 1.27–1.32).

COVID-19 infection was higher among those presenting with clinical symptoms than those who were asymptomatic (OR. 4.52; 95% CI 4.42–4.64), as shown in Table 3. almost the same ORs were provided when the analysis was repeated for each exposure factor adjusting for the remaining factors.

## Discussion

This study provides the epidemiological characteristics of the SARS-CoV-2 outbreak at the primary healthcare level in Qatar, where the PHCC operates. It adds a better understanding of the epidemiology of the pandemic in an ideal demographic setting since it excludes male manual workers registered with Qatar Red Crescent health centers for their primary healthcare services.

In Qatar, testing for SARS-CoV-2 started on February 5, 2020, with the first case identified on February 28, 2020, among returning travelers. During this time, the infection had spread in many countries over the globe. Meanwhile, the State of Qatar established a national plan to respond to the anticipated COVID-19 outbreak. Testing for SARS-CoV-2 was scaled-up as of March 2020 when the first cluster of 300 cases was identified among expatriate workers on March 8, 2020.^17^ The effective and restrictive case identification and contact tracing measures were probably the reasons behind the low number of cases until March 2020. During that time, a substantial number of returning travelers and nationals were identified to have COVID-19 infection.^18,21^

Qatar took a series of public health measures in response to the spread of COVID-19. The measures included limiting incoming passenger flights into Doha through Hamad International Airport and allocating quarantine facilities for returning travelers. Other measures were implemented gradually that promoted using face masks and physical distancing, including closing retail stores and malls, suspending larger sports activities, and enforcing working from home for 80% of the public and private sectors.^18^ The latter measures started to ease based on a phased approach for lifting restrictions from June 15, 2020, onwards.

As of April 2020, the number of daily cases and, subsequently, the positivity rate started to increase steadily at the community level, partly because of the scaled-up capacity of PHCC in conducting screening tests and the expansion of the epidemic in the wider population. The positivity rate reached its highest level in May at 37.5%. The latter might be attributed to the month of Ramadan, where visits and social gatherings around breaking the fast meals are a culturally impeded practice among all people observing Ramadan.

The epidemiological characteristics of the tested persons at the community level through PHCC testing sites for the PHCC-registered population showed that most tested persons were from the Middle East and Northern Africa region at 57.0%, followed by persons originally coming from Asia at 32.5%. The latter reflects the distribution of the registered population at primary healthcare services in Qatar, which reflects the diversity and dynamics of the demographic distribution of the population in the state. In Qatar, 88% of the population are expatriates due to their workforce needs.^11^ Positivity rates among the age-group 60+ years were the lowest at 8.7% compared to other age groups. The latter might be attributed to the effective public health measures that Qatar adopted in response to the COVID-19 pandemic, specifically targeting older adults and high-risk groups.^18^ In addition, the older adults aged 60+ years were a distinctive small proportion of the PHCC-registered population, as shown in Figure 1. Persons who reported clinical symptoms of influenza-like-illness when conducting the COVID-19 test at the PHCC testing sites had a higher COVID-19 infection rate than those who were asymptomatic (OR. 4.52; 95% CI 4.42–4.64).

The most common comorbidities among the persons who were tested for SARS-CoV-2 at the PHCC testing sites were diabetes mellitus (10.9%) and hypertension (11.3%). The latter reflects the noncommunicable diseases prevalence distribution among the PHCC target population. Based on the health needs assessment study conducted in 2019 among the PHCC-registered population, the prevalence of diabetes mellitus ranged between 11.9% and 13.9%, and hypertension between 11.8% and 15.7%.^22^

The highest positivity rate was observed among males from Asian countries at 15.7%. The latter might be attributed to their living conditions in more crowded areas and accommodations. Also, despite the national restrictions, their social behavior of social mixing, limited persons’ movement and gathering except in urgent situations. In a study conducted on COVID-19 infection at workplaces in Qatar, the highest COVID-19 infection positivity rate was found among males from South Asia.^23^

This study applied a multivariable logistic regression model to understand the effect of sex, nationality group, age groups, and symptoms presentation on capturing COVID-19 infection. Sex affected COVID-19 infection, with males having a higher risk than females (OR 1.15; 95% CI 1.13–1.17). In a similar study conducted in Tianjin in China, males were at higher risk of developing severe COVID-19 infection, which highlighted the need for early COVID-19 testing and intervention.^24^

The strengths of this study include all the tested cases at the community level where PHCC had testing sites, with all the tests conducted at a single laboratory. PHCC provided accessibility for COVID-19 testing at the community level by allocating four COVID-19 testing and holding centers and at least one testing room in the remaining 23 health centers. All tests performed at PHCC were included, providing a robust estimation of the positivity rate among those tested. There are limitations to this study. The age-adjusted rates were not calculated. The exact national address was not completely captured in the system, and noncommunicable disease data was not entirely recorded in the patient electronic medical record.

## Conclusion

In conclusion, this study provides detailed information on COVID-19 in Qatar at the primary healthcare level by analyzing the epidemiological characteristics of tested cases among the PHCC-registered population between March 11 and December 31, 2020. The epidemic predominantly affected younger ages and males, namely Asians, matched with the national studies, including the manual workers. At the primary healthcare level, the COVID-19 infection rate was higher among those who presented with clinical symptoms. The high-risk group, the 60+ years group, showed the lowest positivity rate, which reflects the effectiveness of preventive measures related to the high-risk group. The scale-up of the testing at the primary care level in Qatar helped to find more cases and was reflected in a steady increase in the positivity rate during the peak of the first wave.

## Declarations

### Ethics approval

The epidemiological characteristics of the primary healthcare-based COVID-19 swabbed persons in Qatar study was approved by the PHCC scientific research committee with reference number PHCC/DCR/2020/07/076.

### Conflict of interest

The corresponding authors and all the coauthors of this paper declare that they have no conflict of interest.

### Data availability statement

The datasets generated during and/or analyzed during the study are available from the corresponding author on reasonable request.

### Funding

This research received no external funding.

### Acknowledgment

We would like to extend our gratitude for all the frontline healthcare workers at the PHCC health centers for their efforts and dedication throughout the pandemic.

## Figures and Tables

**Table 1:** Characteristics of the swabbed persons at the PHCC health centers between March 11 and December 31, 2020

	Male n (%)	Female n (%)	Total n (%)

Demography			

Mean age, years (SD)	32.3 (17.3)	31.2 (16.4)	31.9 (16.8)

Median age, years (IQR)	32 (20–43)	31 (21–41)	32 (21–42)

0–18 years	43,181 (22.0)	39,542 (21.7)	82,723 (21.8)

19–39 years	90,399 (46.0)	92,180 (50.5)	182,579 (48.2)

40–59 years	49,376 (25.2)	41,441 (22.7)	90,817 (24.0)

60+ years	9,458 (5.2)	13,627 (6.9)	23,085 (6.1)

Nationality-grouped			

Middle East and North African (MENA)	115,293 (58,7)	100,737 (55.2)	216,030 (57.0)

Asian	62,627 (31.9)	60,661 (33.2)	123,288 (32.5)

African	3,810 (1.9)	6,954 (3.8)	10,764 (2.8)

Others	14,853 (7.8)	14,269 (7.6)	29,122 (7.7)

Reason for swabbing			

Clinical suspicion	50,837 (28.9)	51,507 (28.2)	102,344 (27.0)

Individual request	23,676 (12)	20,937 (12.0)	44,613 (11.8)

Survey	24,692 (12.0)	22,250 (11.5)	46,942 (12.4)

Contact tracing	15,530 (7.9)	17,449 (9.6)	32,979 (8.7)

Port of entry	26,150 (13.3)	24,321 (13.3)	50,471 (13.3)

Healthcare routine check up	3,345 (1.7)	4,253 (2.3)	7,598 (2.0)

Others-Not specified	11,594 (5.9)	10,772 (5.9)	22,366 (5.9)

Missing	27,614 (14.1)	19,112 (10.5)	46,726 (12.3)

Symptoms presentation			

Symptomatic	43,943 (26.1)	45,482 (28.0)	89,425 (27.0)

Asymptomatic	124,163 (73.9)	117,162 (72.0)	241,325 (73.0)

Comorbidities			

Diabetes mellitus	23,321 (11.9)	17,965 (9.8)	41,286 (10.9)

Cardiovascular disease	6,464 (3.3)	2,379 (1.3)	8,843 (2.3)

Hypertension	24,689 (12.6)	18,071 (9.9)	42,760 (11.3)

Asthma/Chronic obstructive pulmonary disease (COPD)	17,968 (9.1)	16,169 (8.8)	34,137 (9.0)

Dyslipidemia	21,661 (11.0)	17,134 (9.4)	38,795 (10.2)


**Table 2:** Positivity rate by nationality-grouped, age-group, region and symptoms presentation

	Male n (%)	Female n (%)	Total n (%)

Nationality-grouped			

MENA	12,999 (11.3)	10,966 (10.9)	23,965 (11.1)

Asian	9,796 (15.7)	7,325 (12.9)	17,121 (13.9)

African	431 (11.3)	651 (9.4)	1,082 (10.0)

Others	949 (6.4)	922 (6.5)	1,871 (6.4)

Age-group			

0–18 Years	4,236 (9.8)	4139 (10.5)	8,375 (10.1)

19–39 Years	12,205 (13.5)	10,323 (11.2)	22,528 (12.3)

40–59 Years	6,514 (13.9)	4,603 (11.1)	11,117 (12.2)

60+ Years	1220 (8.9)	799 (8.4)	2,019 (8.7)

Region			

Central	8,077 (12.1)	6,875 (10,3)	14,952 (11.2)

Northern	8,700 (11.7)	7,332 (11.1)	16,032 (11.4)

Western	7,398 (13.3)	5,657 (11.3)	13,055 (12.3)

Symptoms presentation			

Asymptomatic	6,108 (4.9)	5,805 (4.9)	11,913 (4.9)

Symptomatic	8,308 (18.3)	8,717 (19.8)	17,025 (19.0)


**Table 3:** Factors associated with the positivity rate (multi regression model)

Variable	OR	95% CI	P value

Sex			

Female (reference)			

Male	1.15	1.13–1.17	< 0.001

Age-group			

0–18 Years (reference)			

19–39 Years	1.25	1.22–1.28	< 0.001

40–59 Years	1.24	1.20–1.28	< 0.001

60+ Years	0.85	0.81–0.90	< 0.001

Nationality-grouped			

MENA (reference)			

Asian	1.29	1.27–1.32	< 0.001

African	0.90	0.84–0.96	< 0.001

Others	0.55	0.52–0.58	< 0.001

Symptoms presentation			

Asymptomatic (reference)			

Symptomatic	4.52	4.42–4.64	< 0.001

